# Supplementary material for: Comparative genomics provides new insights into the diversity, physiology, and sexuality of the only industrially exploited tremellomycete: Phaffia rhodozyma
Source: BMC Genomics. 2016 Nov 9;17:901. doi: 10.1186/s12864-016-3244-7 (PMC5103461; doi:10.1186/s12864-016-3244-7)
Supplement: Additional file 6: — List of orphan genes with links to PFAM (related to Additional file 1: Table S1). (ZIP 1428 kb) [file 12864_2016_3244_MOESM6_ESM.zip › BLAST_HTML_FTR/G00177_P.html]

BLAST Search Results


```
BLASTP 2.2.27+


Reference:
Stephen F. Altschul, Thomas L. Madden, Alejandro A. Schäffer,
Jinghui Zhang, Zheng Zhang, Webb Miller, and David J. Lipman (1997),
"Gapped BLAST and PSI-BLAST: a new generation of protein database
search programs", Nucleic Acids Res. 25:3389-3402.


Reference for
composition-based statistics:
Alejandro A. Schäffer, L. Aravind, Thomas L. Madden, Sergei
Shavirin, John L. Spouge, Yuri I. Wolf, Eugene V. Koonin, and
Stephen F. Altschul (2001), "Improving the accuracy of PSI-BLAST
protein database searches with composition-based statistics and
other refinements", Nucleic Acids Res. 29:2994-3005.


Database: nr
           71,551,133 sequences; 26,053,659,533 total letters


Query= G00177_P

Length=546
                                                                      Score     E
Sequences producing significant alignments:                          (Bits)  Value

emb|CED84027.1|  hypothetical protein [Xanthophyllomyces dendrorh...  1076    0.0  
ref|XP_011179835.1|  PREDICTED: uncharacterized protein LOC105210...  52.4    0.001


 >emb|CED84027.1| hypothetical protein [Xanthophyllomyces dendrorhous]
Length=545

 Score = 1076 bits (2783),  Expect = 0.0, Method: Compositional matrix adjust.
 Identities = 544/545 (99%), Positives = 545/545 (100%), Gaps = 0/545 (0%)

Query  1    MPPIKRIRIAQPKSDPITTGRGWLLSKIDELEASLKDYVIASGLVLRYHADLLQQQQTKN  60
            MPPIKRIRIAQPKSDPITTGRGWLLSKIDELEASLKDYVIASGLVLRYHADLLQQQQTKN
Sbjct  1    MPPIKRIRIAQPKSDPITTGRGWLLSKIDELEASLKDYVIASGLVLRYHADLLQQQQTKN  60

Query  61   ELSAQLAQPPKELLDRLQQNNYAWIRAELDEYLRLAQTPEQAPEDSTLANTDDQINSSSL  120
            ELSAQLAQPPKELLDRLQQNNYAWIRAELDEYLRLAQTPEQAPEDSTLANTDDQINSSSL
Sbjct  61   ELSAQLAQPPKELLDRLQQNNYAWIRAELDEYLRLAQTPEQAPEDSTLANTDDQINSSSL  120

Query  121  VPSTASATTSAASTSTAQPVPEPVSRQPVVVKNQLTGEHAISAQPSLGQADPFKPAGQSS  180
            VPSTASATTSAASTSTAQPVPEPVSRQP+VVKNQLTGEHAISAQPSLGQADPFKPAGQSS
Sbjct  121  VPSTASATTSAASTSTAQPVPEPVSRQPIVVKNQLTGEHAISAQPSLGQADPFKPAGQSS  180

Query  181  TPSLSSTTPSVPAPVPASAPPPLTVAAAVAAASSTNDHINTINDRDNDGKLNPALSFNPQ  240
            TPSLSSTTPSVPAPVPASAPPPLTVAAAVAAASSTNDHINTINDRDNDGKLNPALSFNPQ
Sbjct  181  TPSLSSTTPSVPAPVPASAPPPLTVAAAVAAASSTNDHINTINDRDNDGKLNPALSFNPQ  240

Query  241  NPHFPRSASTLPGSSMMMTTTEEQPSPVPFVTKPRLKPKPRMKPLLGTMETETEDEMRTS  300
            NPHFPRSASTLPGSSMMMTTTEEQPSPVPFVTKPRLKPKPRMKPLLGTMETETEDEMRTS
Sbjct  241  NPHFPRSASTLPGSSMMMTTTEEQPSPVPFVTKPRLKPKPRMKPLLGTMETETEDEMRTS  300

Query  301  GVGGTRMSSIVDPLLGVVDDSLVQPNMIITPGGGTTRPDGSLASHPSTFFPPPPPSPPLA  360
            GVGGTRMSSIVDPLLGVVDDSLVQPNMIITPGGGTTRPDGSLASHPSTFFPPPPPSPPLA
Sbjct  301  GVGGTRMSSIVDPLLGVVDDSLVQPNMIITPGGGTTRPDGSLASHPSTFFPPPPPSPPLA  360

Query  361  APPVPTPATSLTTTPSSLLQPLPTASSSSAQTQMQMQLPIQMQGFPQTLTAAEIDSMTSE  420
            APPVPTPATSLTTTPSSLLQPLPTASSSSAQTQMQMQLPIQMQGFPQTLTAAEIDSMTSE
Sbjct  361  APPVPTPATSLTTTPSSLLQPLPTASSSSAQTQMQMQLPIQMQGFPQTLTAAEIDSMTSE  420

Query  421  QAQAIMDQLNAAVSASASASAPVLPSAPVSVSQSQSQSQSQPQKPSMSIPIPPMDGVIDL  480
            QAQAIMDQLNAAVSASASASAPVLPSAPVSVSQSQSQSQSQPQKPSMSIPIPPMDGVIDL
Sbjct  421  QAQAIMDQLNAAVSASASASAPVLPSAPVSVSQSQSQSQSQPQKPSMSIPIPPMDGVIDL  480

Query  481  TDESDELNMASTAMNTNTNTNVSANADAGAGTINIETGDMTDLSEFLNDVDGENWQQILN  540
            TDESDELNMASTAMNTNTNTNVSANADAGAGTINIETGDMTDLSEFLNDVDGENWQQILN
Sbjct  481  TDESDELNMASTAMNTNTNTNVSANADAGAGTINIETGDMTDLSEFLNDVDGENWQQILN  540

Query  541  QFGQS  545
            QFGQS
Sbjct  541  QFGQS  545


>ref|XP_011179835.1| PREDICTED: uncharacterized protein LOC105210520 [Bactrocera cucurbitae]
Length=2909

 Score = 52.4 bits (124),  Expect = 0.001, Method: Composition-based stats.
 Identities = 54/224 (24%), Positives = 85/224 (38%), Gaps = 16/224 (7%)

Query  177  GQSSTPSLSSTTPSVPAPVPASAPPPLTVAAAVAAASSTNDHINTINDRDNDGKLNPALS  236
            G  +TP   +T+ S P   P   P P +++   A  S+        + +     + P  +
Sbjct  112  GPFTTPC--TTSASTPNSSPPRTPKPTSLSPTTAPISTPTPTTCDTSTQTPSKPITPKPT  169

Query  237  FNPQNPHFP---RSASTLPGSSMMMTTTEEQPSPVPFVTKPRLKPKPRMKPLLGTMETET  293
             NP+    P   ++ ++ P  +   T +  Q    P   KP   PKP   P      T+T
Sbjct  170  INPRPSSTPTQTQATTSKPCDAPTPTQSSSQTPSKPITPKPTTNPKPSPTP------TQT  223

Query  294  EDEMRTSGVGGT-RMSSIVDPLLGVVDDSLVQPNMIITP----GGGTTRPDGSLASHPST  348
            +  + T+    T   SS   P   +       P    TP       +T  D    +  S+
Sbjct  224  QTPLSTACDTPTPTQSSSQTPSKPITPKPTTNPKPSPTPTQTQSPTSTPCDTPTPTQSSS  283

Query  349  FFPPPPPSPPLAAPPVPTPATSLTTTPSSLLQPLPTASSSSAQT  392
              P  P +P     P P+P  + T TP S     PT + SS+QT
Sbjct  284  QTPSKPNTPKPTKNPQPSPTPTQTKTPLSTACDTPTPTQSSSQT  327


 Score = 40.8 bits (94),  Expect = 5.0, Method: Composition-based stats.
 Identities = 107/452 (24%), Positives = 150/452 (33%), Gaps = 66/452 (15%)

Query  98   TPEQAPEDS---TLANTDDQINSSSLVPS-------TASATTSAASTSTAQPVPEPVSRQ  147
            +P  AP  +   T  +T  Q  S  + P        +++ T + A+TS     P P    
Sbjct  139  SPTTAPISTPTPTTCDTSTQTPSKPITPKPTINPRPSSTPTQTQATTSKPCDAPTPTQSS  198

Query  148  PVVVKNQLTGEHAISAQPS---LGQADPFKPAGQSSTPSLSST-TPSVP-APVPASAPPP  202
                   +T +   + +PS        P   A  + TP+ SS+ TPS P  P P + P P
Sbjct  199  SQTPSKPITPKPTTNPKPSPTPTQTQTPLSTACDTPTPTQSSSQTPSKPITPKPTTNPKP  258

Query  203  LTVAAAVAAASST--NDHINTINDRDNDGKLN-PALSFNPQNPHFPRSASTLPGSSMMMT  259
                    + +ST  +    T +      K N P  + NPQ    P    T   ++    
Sbjct  259  SPTPTQTQSPTSTPCDTPTPTQSSSQTPSKPNTPKPTKNPQPSPTPTQTKTPLSTACDTP  318

Query  260  TTEEQPSPVPFVTKPRLKPKPRMKPLLGTMETETEDEMRTSGVGGT-RMSSIVDPLLGVV  318
            T  +  S  P  +KP + PKP   P      T+TE    T     T   SS   P   + 
Sbjct  319  TPTQSSSQTP--SKP-ITPKPTTNPKPSPTPTQTETPTSTPCDTPTPTQSSSQTPFKPIT  375

Query  319  DDSLVQPNMIITPGG---------GTTRPDGSLASHPSTFFPPPPPSPPLAAPP------  363
                  P    TP            T  P  S +  PS    P P + P  +P       
Sbjct  376  PKPTTNPKPSPTPTQTETPTSTPCDTPTPTQSSSQTPSKPIIPKPTTNPKPSPTPTQTET  435

Query  364  -------VPTPATSLTTTPSSLLQPLPTAS----------------SSSAQTQMQMQLPI  400
                    PTP  S + TPS  + P PT +                 S   TQ   Q+P 
Sbjct  436  ATSTPCDTPTPTQSSSQTPSKPITPKPTTNPKPSPTPTQTQTPTSTPSDTPTQSSSQIPS  495

Query  401  QMQGFPQTLTAAEIDSMTSEQAQAIMDQLNAA-----VSASASASAPVLPSAPVSVSQSQ  455
            +      T       + T  Q     D L            +S  AP   S P S S   
Sbjct  496  KPISPKPTTNPKPSPTPTQRQTPTPSDTLTPTQSSSPTPPKSSTPAPTASSKP-SPSTIP  554

Query  456  SQSQSQPQKPSMSIPIPPMDGVIDLTDESDEL  487
            +Q+       S S P P + G  + T   D L
Sbjct  555  TQTPPNDNIISTSSPTPSIPGPTEETSGCDPL  586


Lambda      K        H        a         alpha
   0.308    0.123    0.346    0.792     4.96 

Gapped
Lambda      K        H        a         alpha    sigma
   0.267   0.0410    0.140     1.90     42.6     43.6 

Effective search space used: 5807756286150


  Database: nr
    Posted date:  Sep 23, 2015 12:05 AM
  Number of letters in database: 26,053,659,533
  Number of sequences in database:  71,551,133


Matrix: BLOSUM62
Gap Penalties: Existence: 11, Extension: 1
Neighboring words threshold: 11
Window for multiple hits: 40
```
